# Early visualization of skin burn severity using a topically applied dye-loaded liquid bandage

**DOI:** 10.1038/s41598-020-65747-x

**Published:** 2020-06-09

**Authors:** John Quan Nguyen, Haley L. Marks, Tyler Everett, Timothy Haire, Anders Carlsson, Rodney Chan, Conor L. Evans

**Affiliations:** 1 Wellman Center for Photomedicine, Massachusetts General Hospital, Boston, 02114 USA; 2 United States Army Institute of Surgical Research, Fort Sam Houston, 78234 USA

**Keywords:** Biomedical engineering, Biophotonics

## Abstract

Skin burns are a significant source of injury in both military and civilian sectors. They are especially problematic in low resource environments where non-fatal injuries can lead to high morbidity rates, prolonged hospitalization, and disability. These multifaceted wounds can be highly complex and must be quickly diagnosed and treated to achieve optimal outcomes. When the appropriate resources are available, the current gold standard for assessing skin burns is through tissue punch biopsies followed by histological analysis. Apart from being invasive, costly, and time-consuming, this method can suffer from heterogeneous sampling errors when interrogating large burn areas. Here we present a practical method for the early visualization of skin burn severity using a topically applied fluorescein-loaded liquid bandage and an unmodified commercial digital camera. Quantitative linear mixed effects models of color images from a four day porcine burn study demonstrate that colorimetric changes within the HSB colorspace can be used to estimate burn depth severity immediately after burning. The finding was verified using fluorescence imaging, tissue cross-sectioning, and histopathology. This low-cost, rapid, and non-invasive color analysis approach demonstrates the potential of dye-loaded liquid bandages as a method for skin burn assessment in settings such as emergency medicine triage and low resource environments.

## Introduction

Skin burns are a significant source of battlefield injury and have accounted for approximately 4% of all combat mortalities since World War II^1–4^. Due to the nature of explosive devices such as landmines, artillery munitions, mortar rounds, and improvised explosive devices, these multifaceted wounds can be highly complex and must be quickly diagnosed and treated in a potentially resource limited environment. In the civilian sector, skin burns are problematic as an estimated 300,000 people world-wide die from fire-related burn injuries every year, with the majority of cases occurring in low and middle income countries^5^. Even when non-fatal, skin burns are a leading cause of future morbidity, prolonged hospitalization, disfigurement, and disability, which can all inadvertently lead to further negative socio-economic repercussions^6^.

Despite a field accuracy of about 50% to 76%, subjective visual and tactile assessments by a trained physician are the most commonly used method for assessing skin burn severity due to their simplicity and speed in clinical settings^7,8^. While useful for evaluating superficial and full-thickness burns, clinical observation is widely considered suboptimal for distinguishing between different types of partial-thickness burns. This is problematic as treatment methods can vary dramatically depending on skin burn severity. Superficial-partial thickness burns can generally heal via natural re-epithelization with minimal scaring while deep-partial thickness burns will often require extensive surgical excision and grafting^9^. Further complicating the issue is that burn wounds can progress in severity during the first 48 hours, and thus it is highly recommended to continuously monitor early-stage burns when deciding optimal treatment^10^. When the appropriate resources are available, the current gold standard for assessing skin burns is through tissue punch biopsies followed by histological analysis^7,10^. Apart from being invasive, costly, time-consuming, and subjective to inter-observer variations between pathologists, histological examination can also suffer from heterogeneous sampling errors when interrogating large area burns^7,11,12^.

Various wide-field non-invasive techniques are currently under development with the aim of augmenting burn wound assessment. Of these, laser Doppler imaging based methods are the most ubiquitous, with several commercial products currently registered under 510(k) premarket submission with the United States Food and Drug Administration^13^. These systems are capable of optically measuring the blood flow within a burned region of skin, which can then be correlated with burn severity. Laser Doppler based methods have consistently demonstrated high sensitivity and specificity varying between 74 and 100% when used to measure skin burns within the first 48 hours^14,15^. Other technologies based on near-infrared spectroscopy, hyperspectral imaging, and spatial frequency domain imaging (SFDI) can provide information about functional tissue health by measuring the changes in clinically relevant chromophores such as blood oxygen saturation and water concentration^11^. In particular, a porcine study using SFDI was able to demonstrate that measurements of tissue oxygenation and tissue scattering related to collagen structure could be used to differentiate superficial-partial thickness burns from more severe burns within the first 2 hours post-burn with tissue scattering showing a strong correlation (r2 > 0.89) to histological burn depth^16^. Despite impressive results, these devices are not without their shortcomings^11^. Each individual device is expensive, physically large, and difficult to position when interrogating non-prominent burn locations such as the perineum. In addition, the time required to position, calibrate, and acquire measurements using these systems can take several minutes, during which the patient must remain motionless to avoid imaging artifacts. These drawbacks can greatly limit the use of such technologies in low-resource military and civilian environments.

Here we present a colorimetric method for rapid, low-cost, and non-invasive visualization of wide-field skin burn severity using a topically applied fluorescein loaded liquid bandage formulation. Color images from a four day porcine burn study in collaboration with the United States Army Institute of Surgical Research were converted into hue, saturation, and brightness (HSB) color space and analyzed to estimate burn depth severity. Colorimetric changes imparted by the bandage onto the skin and measured by an unmodified commercial grade digital camera were found to be predictive of burn depth severity within the first 3 days of burn exposure prior to the onset of visible wound healing. As fluorescein is a fluorescent dye, the outcomes were additionally explored by measuring tissue fluorescence as well as standard histopathology.

## Methods

### Animal

A 3.5 month old cross-bred Yorkshire hybrid female pig (Midwest Research Swine, Gibbon, MN) weighing 50.4 kg was used for this Institutional Animal Care and Use Committee (IACUC) approved study at the United States Army Institute for Surgical Research. Procedures for inducing anesthesia were similar to those described by *Olekson et al*.^17^. On day 0 prior to anesthesia, the pig was pre-medicated with intramuscular injections of glycopyrrolate (0.01 mg/kg) as needed in order to reduce saliva secretion and prevent vagally mediated bradycardia during the procedure. The pig was then induced with an intramuscular injection of tiletamine-zolazepam (Telazol, 4–6 mg/kg) and initially anesthetized with 3–5% isoflurane in oxygen via a facemask. Afterwards, the pig was intubated with an endotracheal tube and placed on a mechanical ventilator with an initial tidal volume of 8–12 ml/kg, peak pressure of 20 cm H_2_O, and respiration rate of 8–20 breaths per minute. The ventilator setting was adjusted to maintain an end tidal PCO_2_ of 40 ± 5 mmHg while anesthesia was maintained with 1% to 4% isoflurane. For each of the following 3 days of the study, the pig was induced with intramuscular injections of tiletamine-zolazepam (Telazol, 4–6 mg/kg) at the left lateral neck and anesthetized initially with 5% isoflurane in oxygen via a facemask before being maintained at 1% to 3% for the rest of the day’s procedures while intubated with an endotracheal tube and placed on a mechanical ventilator. A Foley catheter and ear vein catheter were placed in order to provide fluids and monitor urine output. Analgesia was administered preemptively prior to the initial burn procedure with subcutanoues applications of hydromorphone (0.2–0.6 mg/kg) into the right lateral neck. Throughout the course of the procedures, temperature probes were used to monitor core body temperature, which was maintained with underbody warming pads. Ioban 2 antimicrobial incise drape (3M Company, Maplewood, MN) and a fabric vest was used to provide additional protection and stabilization. In order to monitor the effects of analgesia, the pig was observed for pain or adverse effects twice each day. At the completion of surgery on day 3, the endotracheal tube was removed and the pig was allowed to stabilize under observation. The pig was humanely euthanized with Fatal Plus IV upon completion of the experiment.

Research was conducted in compliance with the Animal Welfare Act, the implementing Animal Welfare Regulations, and the principles of the National Research Council’s Guide for the Care and Use of Laboratory Animals^18^. The facility’s IACUC approved all research conducted in this study. The facility where this research was conducted is fully accredited by the Association for Assessment and Accreditation of Laboratory Animal care (AAALAC).

### Burn model

**Figure 1:**
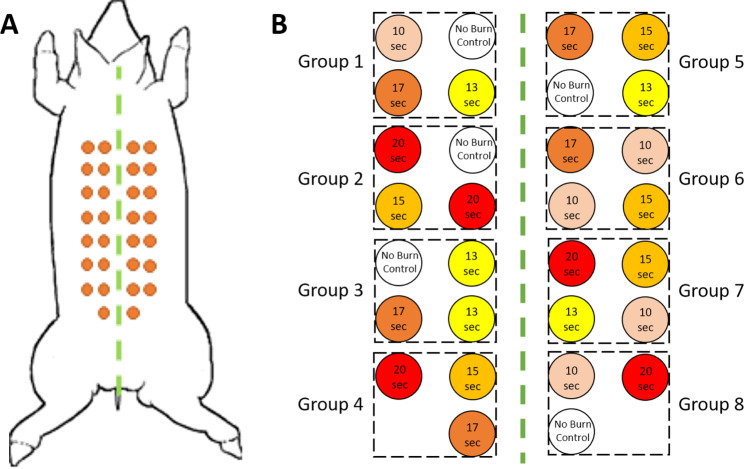
(**A**) Map of burn wound locations on the pig’s dorsum. (**B**) Diagram of each burn wound location and their individual burn times. Black dashed boxes indicate the imaging field-of-view for each group of burn wounds.

Prior to the creation of burn wounds, the dorsum of the anesthetized pig was mechanically shaved, chemically depilated, and washed with sterile water to remove hair. Pre-operative outlines of the intended wound sites were made using a surgical marker upon which a thermocouple-regulated aluminum heating device maintained at 100 °C was applied at a constant pressure in order to induce 3 cm diameter burn wounds similar to a model described by *Singh et al*.^19^. Different burn depth severities were achieved by applying the heating device for 0, 10, 13, 15, 17, or 20 seconds for a total of 30 skin burns with 5 repetitions for each applied burn time. The total wound area accounted for less than 10% of the total body surface area. A map of burn wound locations along with their burn times can be seen in Fig. 1(A,B).

### Experiment timeline

**Figure 2:**
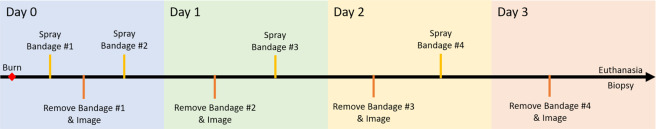
Timeline of the experiment’s procedures. Bandages on day 0 were applied for 20 minutes prior to being removed and imaged. For each of the following days, bandages were applied for 24 hours prior to removal and imaging.

A timeline of the experiment’s procedures can be seen in Fig. 2. On day 0, burn wounds were induced and the the pig was re-stabilized within a span of 45 minutes. The fluorescent liquid bandage solution was then applied to each of the 3 cm diameter burns using a gravity-feed airbrush and compressor (Anest Iwata-Medea, Inc, Portland, USA) with a total of 300 *μ*L of formulation applied across a 4 cm^2^ surface area encompassing each individual burn. A silicone stencil was used to prevent over spraying of the fluorescent liquid bandage to neighboring burn sites. After spraying, the pig’s entire dorsum was covered in a breathable transparent medical dressing (Tegaderm, 3M, St. Paul, MN) for 20 minutes before being removed. Removal of the medical dressing additionally facilitated the removal of the bandage’s thin polymer layer from the skin. Any residual bandage was further removed by gently washing the skin with water and wiping with 70% isopropyl alcohol. The wounds were then imaged and another application of the liquid bandage solution was administered. At the end of the day’s procedures, the pig’s dorsum was once again wrapped in a new layer of medical dressing for 24 hours until the next day’s procedures. A similar procedure of removing the bandages, imaging burn wounds, and reapplication of new bandages and dressings was carried out on each of the following days. After imaging on day 3, the pig was was then euthanized and both punch and cross-sectional biopsies were acquired from each burn wound for histological analysis.

### Histology

Following euthanasia on day 3 of the study, 6 mm diameter punch biopsies were acquired from each burn wound and subsequently formalin fixed, paraffin embedded, and stained with Hematoxylin and Eosin (H&E) for assessment by a certified histopathologist. Cross sectional strip biopsies running lengthwise from dorsal to ventrolateral were also acquired for the estimation of burn depth severity via fluorescence imaging. These strip biopsies were 4 to 8 mm wide and covered the full extent of each burn wound while retaining a small section of control unburned tissue at both ends of the strip biopsy.

### Dye-loaded fluorescent liquid bandage

**Figure 3:**
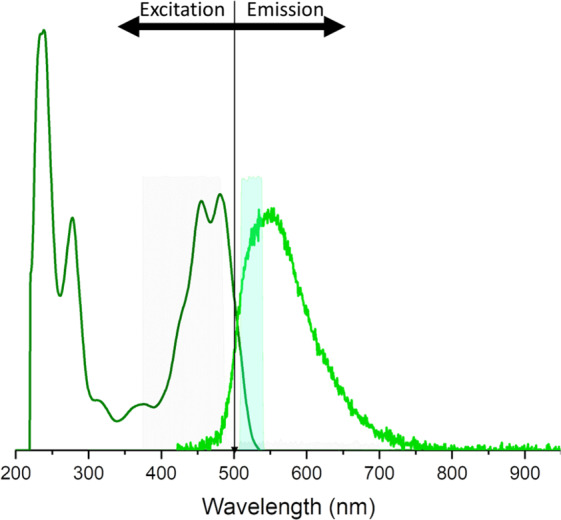
Fluorescence spectra of acidified AK-Fluor in ethanol. The solution used in this study was confirmed to have a doublet excitation peak centered around 470 nm and a peak emission wavelength of 540 nm. The highlighted region represents the bandwidth of the fluorescence imaging camera’s filters.

The transparent liquid bandage formulation was developed to readily adhere to skin for the purpose of burn wound assessment via a simple colorimetric change. This spray-on formulation consists of New-Skin liquid bandage (Advantice Health, LLC, Cedar Knolls, NJ) embedded with a fluorescein reference dye capable of permeating both the epidermal and dermal layers of the skin. The dye-loaded spray-on bandage formulation consists of four components: fast-drying ethanol-based nitrocellulose liquid bandage (New-Skin, Advantice Health LLC, Cedar Knolls, USA), 200 proof ethanol (Sigma-Aldrich, St. Louis, USA) as an airbrushing thinner, an oxygen-sensing porphyrin dendrimer (unused in this study)^20^, and injectable-grade fluorescein dye (AK-Fluor 25%, Akorn Inc., Lake Forest, USA). The dye was first acidified from pH 8.6 to pH 6.8 using 1M HCl and subsequently dissolved in 200 proof ethanol at a concentration of 120 *μ*M. This stock solution was then diluted with the commercial-grade liquid bandage at a ratio of 1:1 to achieve a final fluorescein concentration of ~60 *μ*M. This concentration is approximately 1/4000 the recommended injectable concentration for angiographies and was chosen so that the dye’s emission is visible to a camera under conventional room lighting when painted onto *ex vivo* skin samples with similar autofluorescence properties to the pig used in this study. As confirmed by a spectrometer, this particular fluorescein dissolved in ethanol solution has a broad absorbance spectra with a doublet excitation peak centered around 470 nm and a peak emission wavelength of 540 nm. (Fig. 3).

### Cameras

Color images were acquired using an unmodified commercial off-the-shelf Nikon D3000 (Nikon, Tokyo, Japan) digital single-lens reflex (DSLR) camera equipped with a Nikon AF-S DX Zoom-Nikkor ED 18–55 mm F3.5–5.6G lens (Nikon, Tokyo, Japan). Each color image was acquired with the camera’s built-in flash unit under normal tungsten-halogen operating room lighting with an exposure time of 1/40 sec at 200 ISO, f/5.3, and focal length of 40 mm.

Fluorescence images were acquired using a customized Nikon D3400 (Nikon, Minato, Tokyo, Japan) DSLR Camera equipped with a Nikon AF Micro-Nikkor 60 mm F/2.8D lens (Nikon, Tokyo, Japan) and a pair of Nikon R1C1 Wireless Close-Up Speedlight system for flash illumination (Nikon, Tokyo, Japan). Fluorescein excitation was accomplished by further equipping the flash units with 475 nm short pass OD4 optical filters (Edmund optics, Barrington, USA) while green light emission was collected through an OD6 525/30 nm bandpass filter (Chroma Technology Corp, Bellows Falls, USA). Each fluorescence image was acquired under normal tungsten-halogen operating room lighting with an exposure time of 1/200 sec at 400 ISO, f/3.2, and focal length of 60 mm.

### Analysis

Average values from *en face* circular regions-of-interests (ROIs) encompassing each burn wound were selected for analysis. Raw color images from the commercial off-the-shelf DSLR camera were converted into 24-bit HSB colorspace using the Fiji distribution of ImageJ^21^ with 8-bit color depth assigned to each channel. Unlike the more traditional RGB colorspace which describes visualized color as an additive mixing of three primary components (red, green, blue)^22^, HSB colorspace describes visualized color as changes in hue, saturation, and brightness similar to how humans perceive color-making attributes^23^. In color theory, the hue parameter is described as an angular position within a cylindrical coordinate diagram that is independent of color intensity (chroma) and lightness. It essentially comprises a categorical "pure" color component which can then be further variegated through the other two parameters, saturation and brightness, to create an expansive spectrum of shades and tones. For computer vision and image analysis applications, HSB is advantageous because of its robustness to changes in lighting and shadowing effects that would normally contribute to noise in RGB colorspace^24–26^.

Green fluorescence images from the modified DSLR camera were acquired in raw format and subsequently converted to RGB colorspace for the extraction of only the isolated 8-bit green channel using the rawpy Python library^27^. These images along with their burn wound ROIs were then normalized against the averaged fluorescence intensities of distal unburned skin within the same respective field-of-view in order to correct for system response resulting from potential variations in lighting, imaging distance, angle, and optical aberrations.

Depth of fluorescein dye infiltration within each burn wound was measured from color images acquired from the cross-sectional biopsies. The green channel from each image was isolated in ImageJ and a thresholding macro based on Otsu’s method^28^ was applied that retained portions of the image with intensities larger than 40% of the highest pixel intensity. This portion of the image was then used for determining the average fluorescence depth along with standard deviation.

Statistical analysis and regression modeling was carried out using the R programming language. Linear mixed effects regression (LMER) models were developed for predicting the continuous outcomes of H&E derived burn depth severities based on continuous predictors including hue, saturation, brightness, normalized fluorescence intensity, and study day. These analysis were performed using the lme4^29^ and lmerTest^30^ packages within the R programming language. The following LMER models were developed:

1 $$BurnDepth={B}_{0}+{B}_{1}Day+{B}_{2}Hue+{B}_{3}Saturation+{B}_{4}Brightness+{b}_{0}Group+\varepsilon $$

2 $$BurnDepth={B}_{0}+{B}_{1}Day+{B}_{2}NormalizedFluorescence+{b}_{0}Group+\varepsilon $$

The LMER model presented in Eq. (1) aims to predict burn depth severity based on the longitudinal study day and the unmodified color camera’s HSB measurements. A similar LMER model seen in Eq. (2) was developed for predicting burn depth severity based on the longitudinal study day and the normalized fluorescence measurements from the customized fluorescence imaging camera. In both models, *B*_0_ represents the model’s intercept and *BurnDepth* is defined as a continuous outcome based on input continuous predictors of *Hue*, *Saturation*, *Brightness*, and *NormalizedFluroscence*. *Day* is defined as a fixed effect while *ε* is defined as the residual error. *Group* is defined as the random effect and accounts for any differences in acquisition conditions between each group of four burns. For the null hypothesis to be disproved in either models, the coefficients *B*_1_, *B*_2_, *B*_3_, or *B*_4_ describing the relationships between *BurnDepth*, *Day*, *Hue*, *Saturation*, *Brightness*, and *NormalizedFluroscence* must be significantly greater or less than 0.

## Results

### *En face* imaging

**Figure 4:**
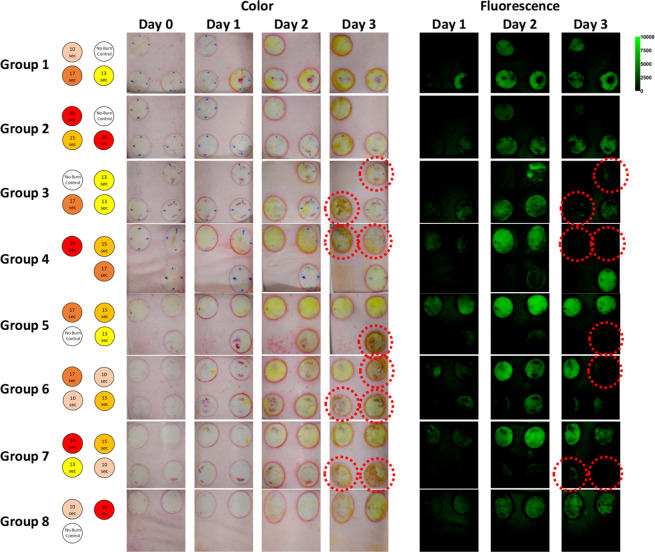
*En-face* color and fluorescence images of each group of burn wounds over each day of the study. Red dashed circles indicate wounds with apparent hypertrophic scar formation.

Thirty circular burn wounds were created on a pig’s dorsum using a 100 °C brass thermocouple device applied at five different durations between 0 to 20 seconds in order to create skin burn severities ranging from superficial to full-thickness with five repetitions for each burn time. For each day of the study, the fluorescein-loaded liquid bandage was sprayed onto each burn wound and then imaged with both a commercial off-the-shelf DSLR camera for the acquisition of color images and a customized DSLR camera for the acquisition of fluorescence intensity images. *En face* color and fluorescence intensity images for each group of burn wounds across each day of the study can be seen in Fig. 4.

Each burn wound exhibits visual characteristics indicative of local response such as a mottled white appearance in regions that were in direct contact with the thermocouple-regulated heating device. Surrounding the perimeter of each burn wound is a ring of increased vascular permeability known as a zone of hyperemia^31^. Due to technical issues, fluorescence images from day 0 were not acquired. Regardless, it can be observed that liquid bandages applied on day 0 imparted a slight greenish coloration onto each burn wound that was not distinguishable from each other by naked eye observation. However for each of the following days, noticeable differences could be observed with the greatest coloration occurring on day 2. Similar changes in green fluorescence intensities could also be observed. By the third and final day of the study, visual indicators of wound healing such as hypertrophic scar formation could be observed in the color images. These same burn wounds exhibited greatly decreased signals in their corresponding fluorescence images, as scar formation is thought to limit dye illumination and excitation.

### Histological validation of burn model

**Figure 5:**
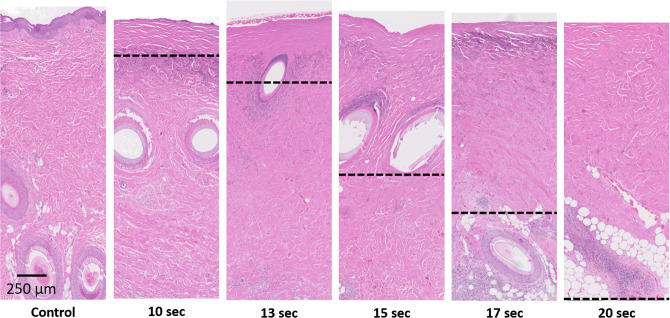
Representative H&E stained punch biopsy samples for each burn time. Dotted Lines represent the estimated burn depth.

The porcine skin burn model was post-operatively validated on the final day of the study through the collection of punch and cross-sectional biopsies of each burn wound. Biopsy samples were stained with hematoxylin and eosin (H&E) and assessed by a practicing medical pathologist in order to determine the final gold-standard burn depth severities based on collagen damage and destruction of adnexal structures such as hair follicles and sweat glands. Example images of histology along with estimated burn depths can be seen in Fig. 5.

**Figure 6:**
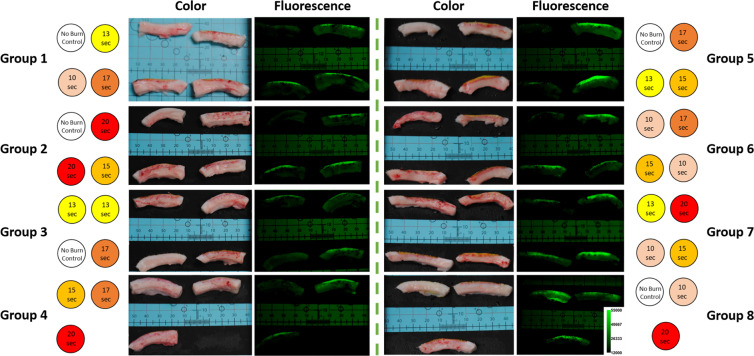
Color images of the post-operative cross-sectional biopsies along with their corresponding fluorescence images.

In addition to H&E histology, the cross sectional biopsies were measured using the fluorescence imaging DSLR camera in order to determine the depth of fluorescein dye uptake within each burn wound. These images can be seen in Fig. 6.

**Figure 7:**
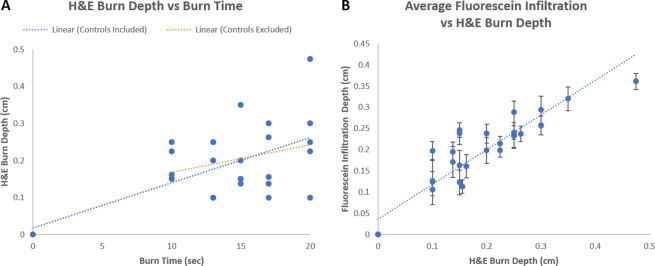
(**A**) Plot of H&E determined burn depths vs applied burn times. The blue dashed line represents a linear fit that includes unburned control samples with R^2^ = 0.50 and p-value = 1.40E-05 while the orange dashed line represents a linear fit that excludes the unburned control samples with R^2^ = 0.084 and p-value = 0.16. (**B**) Plot of average fluorescein infiltration depth vs H&E determined burn depth. Error bars represent standard deviations and dashed lines represents a linear fit with R^2^ = 0.85 and p-value = 3.71E-13.

The plots seen in Fig. 7 illustrate the correlation between the applied burn times, the H&E determined burn depths, and the fluorescein infiltration depth. As seen from the blue dashed line in Fig. 7(A), a significant linear relationship (p-value = 1.40E-05) was initially observed between the applied burn times and the H&E determined burn depths when the unburned control samples were included in analysis. However upon closer inspection, it became apparent that the correlation was biased due to the asymmetrical distribution of measurements skewed by the unburned control samples. Upon their removal from analysis (orange dashed line), the correlation between the applied burn times and H&E derived burn depths were no longer significant (p-value = 0.16) and suggests that the porcine burn model utilized in this particular study did not result in the consistent production of increasing burn depth severities with increasing applied burn times. Despite a lack of correlation between applied burn times and burn depth severities within the context of this study, when the fluorescein infiltration depths were correlated with the H&E determined burn depths as seen in Fig. 7(B), a strong linear relationship was found (p-value = 3.71E-13, R^2^ = 0.85) which suggests that the amount of fluorescein dye infiltration into each burn wound is significantly associated with the amount of structural damage as determined by gold standard H&E histology.

### Statistical analysis

#### Colorimetric changes at early time points are predictive of burn severity

**Figure 8:**
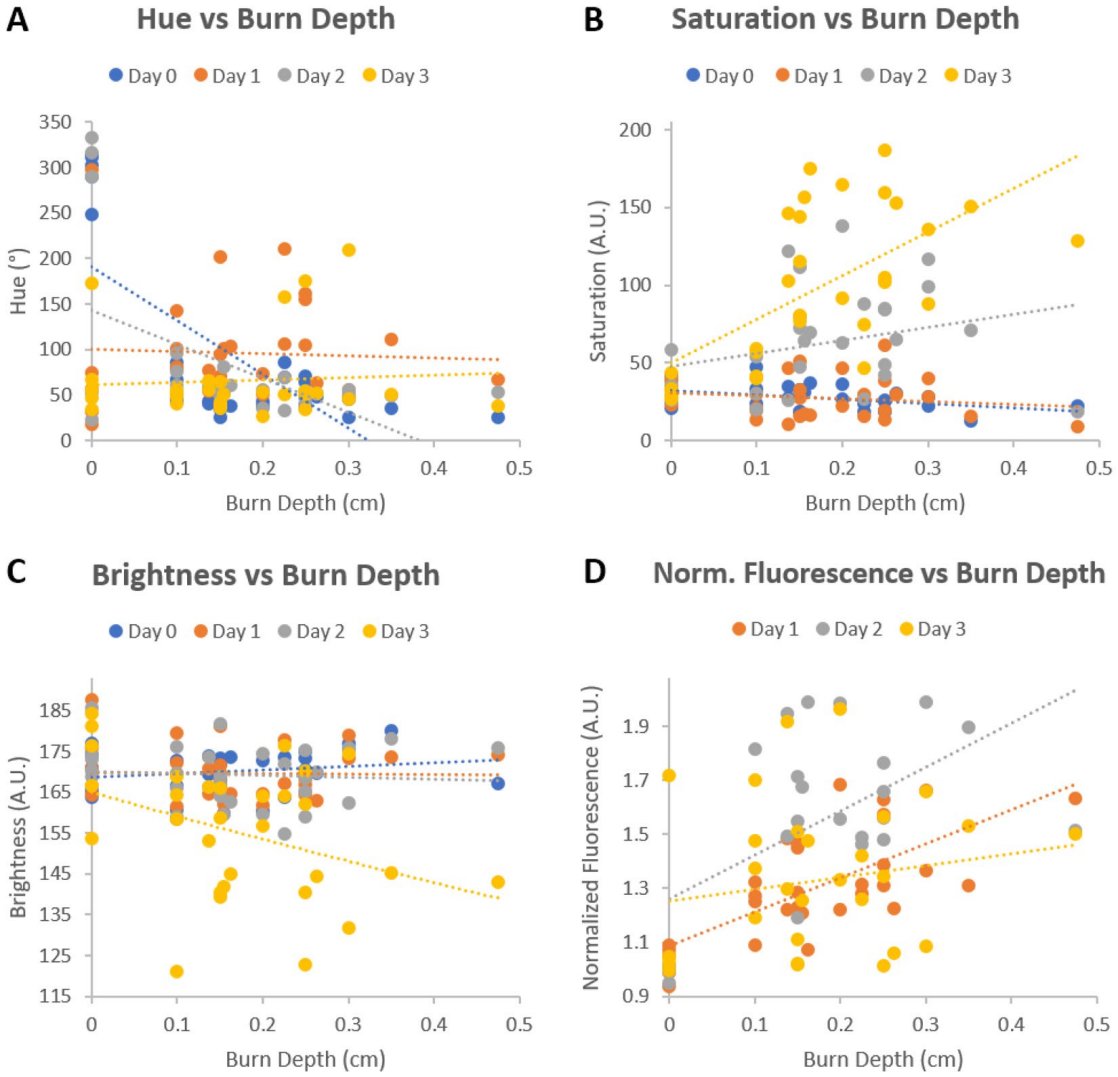
(**A**–**C**) Plots of *en face* HSB measurements vs H&E derived burn depth. (**D**) Plots of *en face* normalized fluorescence intensities vs H&E derived burn depth. Data points represent the mean values within a ROI encompassing the entire burn wound. Dashed lines indicate linear fits. Significance of fits can be seen in Table 1.

A simple linear regression was applied to the daily *en face* camera based measurements of HSB and normalized fluorescence intensities in relation to the H&E derived burn depths. Plots of these relationships can be seen in Fig. 8 and the significance of their correlations can be seen in Table 1.

**Table 1:** Table of p-values resulting from the daily linear correlations of HSB and normalized fluorescence intensities with H&E determined burn depths.

Day	p-values			
	Hue	Saturation	Brightness	Fluorescence Intensity
0	1.14E-05***	0.022*	0.28	N/A
1	0.81	0.41	0.92	1.75E-05***
2	0.0030**	0.12	0.70	0.0046**
3	0.011*	2.1E-04***	0.036*	0.37

Significance codes: <0.001***, <0.01**, and <0.05*.

From these plots, it can be observed that the changes in tissue color visually observed in Fig. 4 are due to specific changes within the HSB colorspace. In particular, the degree of hue was observed to decrease from normal skin tones towards greener hues with increasing burn depth severities. The change in color saturation was also observed to increase with burn depth severity. These trends are consistent with the increasing fluorescence intensities observed which points towards a higher infiltration of fluorescein into deeper more severe burns. Brightness, a value most associated with shadowing artifacts, does not exhibit a clear trend.

From Table 1, it can be observed that colorimetric changes in the hue parameter are significantly correlated with burn depth severities on day 0 (p-value ≪ 0.001), day 2 (p-value < 0.01), and day 3 (p-value < 0.05) of the study with the highest correlation occurring on day 0, 20 minutes after the burns were created. While a positive trend was observed in the saturation plots seen in Fig. 8(B) along with a slight significance on day 0 (p-value < 0.05), none of the other daily changes were found to be significant until day 3 of the study (p-value ≪ 0.001), which was also observed to be a slightly significant timepoint for brightness (p-value < 0.05). However, as visual indicators of wound healing such as eschar formation had already begun on day 3 as seen in Fig. 4, it is difficult to ascertain if the colorimetric changes observed on day 3 were solely from the liquid bandage or from the wound healing process. Fluorescence intensity changes were observed to be significant on day 1 (p-value ≪ 0.001) and day 2 (p-value < 0.01) which further support the trends observed in Fig. 8.

#### LMER models for predicting burn depth severity

As changes in *en face* HSB and normalized fluorescence were observed to be significantly correlated with burn depth severities, these parameters along with the longitudinal study day were utilized as continuous predictors of burn depth within the LMER models presented in Eq. (1) and Eq. (2). The coefficients and their respective p-values generated for Eq. (1) can be seen in Table 2 while those for Eq. (2) can be seen on Table 3.

**Table 2:** Estimates for the LMER model presented in Eq. (1) for predicting burn depth severity based on HSB measurements from an unmodified DSLR camera.

LMER Eq. 1		
Fixed Effect	Estimate of Coefficient	p-value
*B*_0_ (Intercept)	0.26	0.12
*B*_1_ (Day)	−219.30	0.034*
*B*_2_ (Hue)	−5.80E-04	9.38E-06***
*B*_3_ (Saturation)	6.56E-04	0.046*
*B*_4_ (Brightness)	−1.28E-04	0.89

Significance codes: <0.001***, <0.01**, and <0.05*.

**Table 3:** Estimates for the LMER model presented in Eq. (2) for predicting burn depth severity based on normalized fluorescence measurements from a DSLR camera customized for fluorescence imaging.

LMER Eq. 2		
Fixed Effect	Estimate of Coefficient	p-value
*B*_0_ (Intercept)	−0.034	0.61
*B*_1_ (Day)	−0.0019	0.88
*B*_2_ (Normalized Fluorescence)	0.15	1.10E-04***

Significance codes: <0.001***, <0.01**, and <0.05*.

When using an unmodified DSLR camera for imaging burn wounds stained by the fluorescein loaded liquid bandage, the coefficients seen in Table 2 associated with longitudinal study day (p-value < 0.05), hue (p-value ≪ 0.001), and saturation (p-value < 0.05) were found be significant for predicting burn depth severity with hue exhibiting the highest significance. In particular, the model suggests that the depth severity of burn wounds is negatively correlated to both hue and the longitudinal study day while being positively correlated to saturation. For verification purposes, a separate LMER model aimed at predicting burn depth severity based on normalized fluorescence intensities measured from a customized DSLR camera was also generated from which a highly significant positive correlation (p-value ≪ 0.001) was observed as seen in Table 3. Both the coefficients estimated in Tables 2 and 3 are consistent with the trends observed in Fig. 8. Residuals from each model passed the Shapiro-Wilk test for normalcy. Alternative models incorporating interaction terms did not improve the model fit, nor were the interaction terms found to be statistically significant.

### DoD disclaimer

The views expressed in this article are those of the authors and do not reflect the official policy or position of the U.S. Army Medical Department, Department of the Army, DoD, or the U.S. Government.

## Discussion

Skin burns are a significant source of injury in both military and civilian sectors^1–5^. They are especially problematic in low resource environments where even non-fatal burns can lead to higher morbidity rates, prolonged hospitalization, and disability along with further negative socio-economic repercussions^6^. These multifaceted wounds can be highly complex and therefore must be quickly diagnosed and treated before their severity progresses.

Here we present the results from a four day porcine skin burn study in collaboration with the United States Army Institute of Surgical Research which suggests that a topically-applied fluorescein-loaded liquid-bandage could be utilized for early wide-field visualization of skin burn severity through simple colorimetric analysis of images acquired from a commercial off-the-shelf DSLR camera. The results of our study suggests that colorimetric changes within the HSB color space imparted onto the burn area by the dye loaded liquid bandage can be used to assess burn depth severity within four days post-burn. In particular, changes in hue seen in Fig. 8 and Table 1 were observed to be highly significant (p-value ≪ 0.001) immediately 20 minutes after burn injury on day 0 and continued to be significant on days 2 (p-value < 0.01) and day 3 (p-value < 0.05). As burn depth severity increases, the degree of hue decreases towards greener values. This is consistent with both the increasing depth of fluorescein uptake presented in Fig. 6 and significantly increasing *en face* fluorescence intensities shown in Fig. 4 and Table 1 which potentially derives from the increasing fluorescein levels as burn severity increases.

Using our data set, we were also able to develop a LMER model seen in Eq. (1) that could be used to predict the continuous outcomes of burn depth severity based on changes within the HSB colorspace as measured by an unmodified DSLR camera across various days post-burn. Fixed effects with the most significant influence on predicting burn depth severities were the longitudinal study day (p-value < 0.05), hue, (p-value ≪ 0.001), and saturation (p-value < 0.05). Interestingly while the correlation between burn depth and saturation by itself was not originally observed to be highly significant in the daily linear correlations seen in Table 1, its significance in the LMER model suggests a dependent relationship between hue and saturation across each day that could be used to predict burn depth severities. For validation purposes, another LMER model seen in Equation (2) was developed that aims to predict burn depth severity based on daily changes in normalized fluorescence intensities as measured by the customized DSLR camera previously described in our methods. From the results in Table 3, it can be seen that the positive coefficient for normalized fluorescence was highly significant (p-value ≪ 0.001) for predicting burn depth severity. As a whole, our results suggest that deeper burn depth severities mediate higher fluorescein dye uptake which can be measured as changes within the HSB color space by an off-the-shelf unmodified DSLR camera.

Our histological data confirms the well-understood burn-related changes to skin where higher severity burn wounds exhibit deeper and more drastic thermal denaturation of the protective stratum corneum and epidermis along with the organelles, heterogeneous structures, and collagen matrix that comprise the dermis^32^. Additionally, damage to capillary networks that perfuse the skin can lead to a loss of blood flow and the "blanched" appearance that characterizes burned skin. This structural damage along with the loss of perfusion allows for the greater overall accumulation and deeper diffusion of the dye into the burn wounds^33–35^. As exemplified in Fig. 5, the unburned control samples were observed to have an intact stratum corneum barrier as well as layers of viably perfused epidermal and dermal tissue underneath. As such, the penetration of the fluorescein small cationic dye into undamaged tissue (Fig. 6) resulted in an expectedly minimal accumulation after 24 hours of initial application. In contrast, the substantially compromised barrier functions of the full-thickness burn samples helped to augment the accumulation of fluorescein within the damaged epidermis and beyond. For partial-thickness burns, the weaker fluorescence signals observed within the undamaged portions of skin seen in Fig. 6 suggest that while the fluorescein dye is able to infiltrate into deeper undamaged tissues, appreciable accumulation only occurs within the damaged regions where compromised structural and perfusion functionality prevents the rapid clearance of dye into the bloodstream.

As far as we are aware, our work demonstrates the first use of topically applied fluorescein for the purposes of skin burn assessment. Prior work by other groups have shown the feasibility of perfusion based angiograpy in combination with injectable fluorescent dyes such as indocyanaine green (ICG) to varying degrees of success^36–42^. The results of this study suggests that a topically applied dye-loaded solution could be used with any commercial grade camera to assess skin burn severity. Though not tested in this study, the magnitude of the observed color changes suggest that smartphone camera and even visual naked-eye inspection by trained individuals may also be possible for burn severity assessment.

Although these results are encouraging, there are several limitations that will need to be addressed in future studies. First, while the utilized porcine burn model is well-accepted for creating burn wounds of increasing severity based on applied burn time^19^, no such relationship was observed during the course of our particular study. We believe that this may be due to an early onset of wound healing mediated by the use of the liquid bandage vehicle and inadvertent debridement during the removal of medical dressings and bandages prior to imaging. Evidence of early wound healing can be seen in the *en face* color images of Fig. 4 where hypertrophic scar formations can be observed on the final day of the study. This suggests that the severity of each individual burn wound may have evolved throughout the course of the study and that the histological samples acquired on day 3 may not be fully representative of each burn’s dynamic nature. Regardless of the applied burn times, the resulting burn depth severities as determined by gold standard histopathology still allowed for the careful assessment of its relationship to dye uptake as seen in Fig. 7(B). Apart from verifying reproducibility in a larger sample size of pigs, the applicability of the dye loaded liquid bandage approach for burn wound assessment will need to be tested in more realistic and complex scenarios. Future studies will require the use of more combat-relevant burn models where the burn topology is not known immediately following injury. Predictions made using the liquid bandage approach could then be directly tested against biopsy and histological analysis which would provide the critical information needed to potentially improve the bandage formulation and move towards clinical studies.

The rapid, non-invasive, and relatively low cost nature of this dye loaded liquid bandage approach makes it highly suited for skin burn assessment in emergency room, ambulatory, and low resource environments. Its robustness and potential compatibility with ubiquitous smartphone cameras could be applied to polytrauama where burn topology can cover large portions of the body that would make traditional burn assessment difficult. In particular, the potential to assess burn wound severities and outcomes at very early time points makes this approach a simple, inexpensive, and lightweight solution for the rapid triage of burn injuries. Such an approach utilizing a dye-loaded bandage and smartphone camera could find beneficial use within military settings such as prolonged field care scenarios where wounded warriors may require medical care for up to 72 hours before medical evacuation^43^. Additionally, this bandage approach may also be useful in the assessment of victims for medical triage in mass casualty events.
